# Predictors of activity level and retention among African American lay health advisors (LHAs) from The National Witness Project: Implications for the implementation and sustainability of community-based LHA programs from a longitudinal study

**DOI:** 10.1186/s13012-016-0403-9

**Published:** 2016-03-22

**Authors:** Rachel C. Shelton, Sheba King Dunston, Nicole Leoce, Lina Jandorf, Hayley S. Thompson, Danielle M. Crookes, Deborah O. Erwin

**Affiliations:** 1 Department of Sociomedical Sciences, Mailman School of Public Health, Columbia University, 722 168th Street, Room 941, New York, NY 10032 USA; 2 Present Address: Office of Research and Methodology, Question Design Research Laboratory National Centers for Health Statistics, Centers for Disease Control and Prevention, 3311 Toledo Road, Hyattsville, MD 20782 USA; 3 Department of Biostatistics, Mailman School of Public Health, Columbia University, 722 168th Street, New York, NY 10032 USA; 4 Department of Oncological Sciences, Icahn School of Medicine at Mount Sinai, 1 Gustave L. Levy Place, Box 1130, New York, NY 10029 USA; 5 Department of Oncology, Karmanos Cancer Institute, Population Studies and Disparities Research Program, Wayne State University School of Medicine, 4100 John R-MM03CB, Detroit, MI 48201 USA; 6 Department of Epidemiology, Mailman School of Public Health, Columbia University, 722 168th Street, New York, NY 10032 USA; 7 Roswell Park Cancer Institute, Office of Cancer Health Disparities Research, Cancer Prevention & Population Sciences, Elm & Carlton Streets, Buffalo, NY 14263 USA

**Keywords:** Lay health advisors, African Americans, Cancer screening, Sustainability, Implementation, Evidence-based programs

## Abstract

**Background:**

Lay health advisor (LHA) programs are increasingly being implemented in the USA and globally in the context of health promotion and disease prevention. LHAs are effective in addressing health disparities when used to reach medically underserved populations, with strong evidence among African American and Hispanic women. Despite their success and the evidence supporting implementation of LHA programs in community settings, there are tremendous barriers to sustaining LHA programs and little is understood about their implementation and sustainability in “real-world” settings. The purpose of this study was to (1) propose a conceptual framework to investigate factors at individual, social, and organizational levels that impact LHA activity and retention; and (2) use prospective data to investigate the individual, social, and organizational factors that predict activity level and retention among a community-based sample of African American LHAs participating in an effective, evidence-based LHA program (National Witness Project; NWP).

**Methods:**

Seventy-six LHAs were recruited from eight NWP sites across the USA. Baseline predictor data was collected from LHAs during a telephone questionnaire administered between 2010 and 2011. Outcome data on LHA participation and program activity levels were collected in the fall of 2012 from NWP program directors. Chi-square and ANOVA tests were used to identify differences between retained and completely inactive LHAs, and LHAs with high/moderate vs. low/no activity levels. Multivariable logistic regression models were conducted to identify variables that predicted LHA retention and activity levels.

**Results:**

In multivariable models, LHAs based at sites with academic partnerships had increased odds of retention and high/moderate activity levels, even after adjusting for baseline LHA activity level. Higher religiosity among LHAs was associated with decreased odds of being highly/moderately active. LHA role clarity and self-efficacy were associated with retention and high/moderate activity in multivariable models unadjusted for baseline LHA activity level.

**Conclusions:**

Organizational and role-related factors are critical in influencing the retention and activity levels of LHAs. Developing and fostering partnerships with academic institutions will be important strategies to promote successful implementation and sustainability of LHA programs. Clarifying role expectations and building self-efficacy during LHA recruitment and training should be further explored to promote LHA retention and participation.

## Background

Programs and policies that support the use of community-based lay health advisors (LHAs) hold tremendous promise for reducing cancer disparities. LHAs are trained peers or community members who share similar social, economic, cultural, and linguistic characteristics with the population of interest and typically deliver health education, navigation, and support in a range of community-based and clinical settings [1–3]. LHAs are often referred to as promotoras(es), peer educators, community health advisors, navigators, or peer outreach workers in the literature. Such programs are based on the premise that engaging community members contributes to community empowerment and capacity building, while also raising awareness of health and social justice issues, enhancing access to care, and improving health behaviors and outcomes [4]. LHA programs are increasingly being implemented in the USA and globally for a wide range of health issues [2-5]. Research suggests that LHA programs are effective in improving behavior change in several areas, including cancer screening [1, 6–13], with the strongest evidence among racial/ethnic minority women [6, 11, 14–20] who experience greater structural barriers to healthcare [21].

Eliminating racial and ethnic cancer disparities will require the successful dissemination, implementation, and sustainability of culturally appropriate evidence-based programs. Breast cancer in particular is responsible for a large proportion of cancer-related morbidity and mortality among African American women [22].The National Witness Project (NWP) is one example of an evidence-based LHA program; a national replication trial found that NWP was highly effective in increasing breast and cervical cancer screening among African American women [23, 24]. NWP uses a robust theory-based, culturally appropriate model [25] that is comparable to many other community-based LHA programs; during 60–90 min group-based “sessions” in community settings, a minimum of three to four trained African American LHAs provide resources, support, navigation, and education to African American women [20, 26]. Overall, half of the LHAs are African American breast and cervical cancer survivors who deliver empowering testimonials and narratives and serve as “role models” [26–30]; faith-based elements are often included (e.g., hymns, prayers), reflecting the common value of spirituality among African Americans [31–33]. While LHAs are often volunteers, some sites provide LHAs with stipends. LHAs work together with project directors from their site to organize and lead sessions and recruit participants, while project directors help identify host institutions, ensure a network with screening resources, secure funding and partnerships, and recruit LHAs. Project directors may be paid staff or volunteers, depending on program funding. Since 1990, NWP has been replicated and implemented in over 40 sites nationally (in both urban and rural settings), with over 400 volunteers, reaching more than 15,000 women annually. NWP has also been identified as one of the National Cancer Institute’s “Research Tested Intervention Programs” [25].

There are tremendous barriers to sustained implementation of LHA programs in practice, and little is understood about their sustainability. Sustainability has been defined as the continued use of program components and activities for the continued achievement of desirable program and population outcomes [34]. Documented barriers to sustainability of LHA programs include lack of funding and/or limited funding sources, lack of national standards and policies to guide program implementation, difficulty conducting ongoing program evaluation to support program continuation [35–37], and numerous costs to implementing programs (e.g., time and resources related to training, materials, supervision, space, evaluation) [35].

Another challenge to sustaining programs relates to the recruitment and retention of LHAs, as LHAs are often volunteers that receive no financial benefits or receive small stipends [35–38]. There can be high turnover and low activity levels and retention among LHAs, with global attrition rates (i.e., loss of trained eligible pool of LHAs) ranging from 3.2 to 77 % [39, 40]. There are several potential explanations for such variability in participation, including a range of support and incentive structures provided. Attrition rates are particularly high among volunteer LHAs [39–41]. Consistent with the broader literature on the sustainability of evidence-based programs [42], the dropout and low participation of LHAs hampers the impact and sustainability of LHA programs [40, 43, 44].

Studies in this area have been primarily conducted among low- and middle-income countries globally (e.g., [45, 46]). Qualitative and quantitative research suggests that social prestige, financial incentives, community and family approval, and sense of social responsibility and values are key motivating factors that affect LHA performance, activity level, and retention [39, 41, 45–48]. Aspects of the work environment, including clarity of job expectations and organizational processes and practices (incentives, supervision, support, training, etc.) [47, 49, 50], are also likely to influence LHA participation.

The factors influencing the activity level and retention of LHAs likely differ across diverse settings (e.g., US context vs. low- and middle-income countries). No studies, to our knowledge, have examined these factors among African American LHA programs in the USA, and few studies have empirically investigated a range of factors across individual, social, and organizational levels to understand LHA participation. This study seeks to address that gap by investigating individual, social, and organizational factors that predict activity level and retention among a community-based sample of African American LHAs. This research can inform strategies to successfully recruit, train, support, and sustain LHAs in community-based settings, with the ultimate goal of improving the implementation and sustainability of effective LHA programs.

### Conceptual framework

**Fig. 1:**
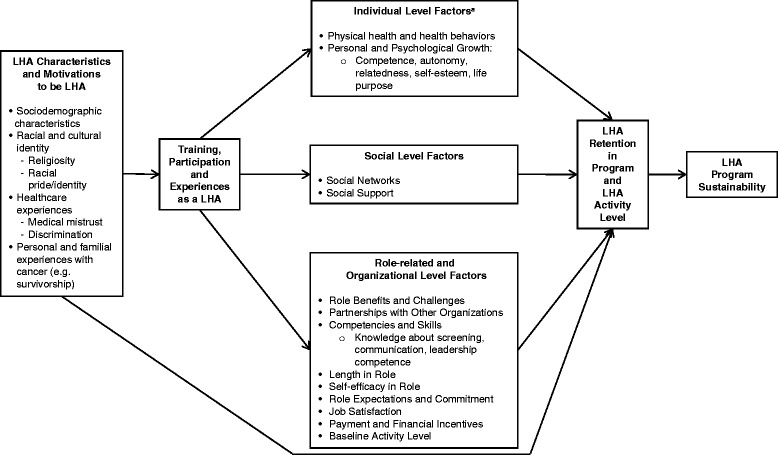
A conceptual framework of factors that impact lay health advisor retention and program activity level. ^a^Individual Level Factors that influence LHA retention and activity level also include LHA Characteristics and Motivations to be LHA

Our conceptual framework (see Fig. 1) for examining factors that predict LHA activity level and retention was informed by prior research and conceptual models that have examined factors associated with retention among LHAs (predominately in global settings) [39, 41, 46, 47, 49, 51], as well as conceptual frameworks that focus on the sustainability of evidence-based interventions [34, 42, 52, 53].

#### Individual level

We examined sociodemographic characteristics that theoretically could impact retention and activity level, including paid employment, age, education, marital status, and general health. We also examined aspects of personal and psychological growth (i.e., competence, autonomy, relatedness, self-esteem, life purpose) that LHAs could experience through the program and might impact their participation. These factors may be particularly important in the context of community-based programs that provide little economic compensation. Though not previously explored, pride in one’s *racial identity* (the extent to which being a member of a specific racial/ethnic group is a significant aspect of one’s identity) [54], *perceptions of discrimination in healthcare* settings and *medical mistrust* [55], and other prior personal experiences or beliefs (e.g., experiences as a *cancer survivor*, *religiosity*) may also provide initial and ongoing motivation to be a LHA in the African American community.

#### Social level

LHA programs rely on a strategy that builds upon and expands existing social networks. LHAs are often trained with other LHAs and work as a team to conduct educational sessions. Further, for many LHA programs, part of their role is to educate and provide support to people within their social networks. Thus, participation in LHA programs may increase the size and composition of one’s *social network* and enhance levels of perceived support. *Providing support* has health-related benefits and is a strong predictor of self-reported health [56, 57]. This is consistent with Riessman’s Helper Therapy Principle (the personal growth and benefits that nonprofessional helpers may experience through their training and service to others) [58] and supports the notion that LHAs may receive social and psychological benefits from their work that motivate them to participate and remain in such programs.

#### Role-related factors and organizational level

*Role-related benefits* experienced (e.g., social recognition, professional development) [49, 59], possible *role challenges* experienced (e.g., burnout, emotional stress, family conflict), and conditions of the work environment (e.g., insufficient training, supervision and leadership, organizational support, resources) could impact retention [49, 60, 61]. LHAs at sites with *partnerships with other organizations that facilitate access to organizational resources pertinent to screening* (e.g., academic institutions, medical centers, hospitals, cancer centers) may also impact participation. LHAs may develop new capacities and transferable *competencies and skills* (e.g., *knowledge* of screening, communication, *leadership competence*), factors that may influence their effectiveness, and potentially retention. *Length in role*, *self-efficacy in role*, *role clarity and commitment*, and *job-satisfaction* are other job-related factors that have not been well examined in this literature but are likely important in understanding retention [38, 62, 63]. Finally, *payment or financial incentives* have consistently been found to be strongly associated with activity level and retention in global settings [40, 47, 49].

## Methods

### Recruitment

We contacted eight NWP sites in the northeast, south, and mid-west regions of the USA. Sites were selected from a total of 20 sites that had attended the most recent NWP Annual Meeting for Education and Networking (AMEN) in 2010. The NWP local project director and the study principal investigator (RS) informed LHAs about the study through a mailed letter, telephone, and presentations at scheduled meetings and trainings. LHAs interested in participating provided written permission to be contacted by the study team. LHAs who expressed interest in participation were contacted by telephone to complete the informed consent process and to schedule the telephone-based study assessment. Institutional Review Board approval was awarded through Columbia University’s Mailman School of Public Health. A total of 84 eligible LHAs provided their contact information (representing the entire pool of eligible LHAs at those sites), and of those, a total of 76 women completed the consent process and participated in the study (response rate = 91 %).

### Eligibility and data collection

Eligible participants had to be self-identified as African American or black, female, a LHA from the NWP (currently or within the past 2 years), over the age of 18, and English-speaking. Predictor variable data were collected during baseline interviewer-administered telephone surveys that took place between 2010 and 2011. Follow-up outcome data on LHA participation and activity levels were collected from project directors in late fall of 2012 and assessed LHA participation for the prior year. All study participants received a $25 gift card for their participation for each interview.

### Predictors

#### Individual-level factors

Sociodemographic information collected from participants included age, income, education, insurance, healthcare provider, and employment. *Autonomy*, *competence*, *and relatedness* were measured using the 20-item Basic Psychological Needs Scale [64] (*α* = .61). *Life engagement* was measured with a six-item scale [65], for which participants rated how much they agreed with each item using a 5-point Likert scale (*α* = .51). *Self-esteem* was measured using the six-item Rosenberg self-esteem scale [66] (*α* = .70). *General self-rated health* was measured with a one-item validated measure [67]. *Breast and cervical cancer survivorship* was determined based on their NWP role (women who have a history of breast or cervical cancer act as both LHAs and “role models” and provide testimonials). *Racial pride* was evaluated using a seven-item scale developed for African Americans [31] (*α* = .80). *Medical mistrust* was measured using a sub-scale on disparities from the Group-Based Medical Mistrust Scale [68] (*α* = .89). *Discrimination in healthcare* was measured with one question from The Experiences of Discrimination Measure [55]. Participants were given a score of 0–3 based on their responses (response options: never, one to two times, three to four times, or five or more times). *Religiosity* was measured using a nine-item scale validated for African Americans [31] (*α* = .86).

#### Social factors

*Social networks* were measured using Cohen’s Social Network Index [69]. This index asked participants about 12 different types of social relationships and the quantity of social ties. For each type of relationship where the participant indicates that they speak to someone (in person or over the telephone) at least once every 2 weeks, that network was assigned 1 point (maximum score = 12). The total number of members in each network was summed to include the number of people they speak to at least once every 2 weeks. *Social support* was measured using the Social Provisions Scale, a validated ten-item scale [56]; respondents rated each item on a scale of 1 to 4 (*α* = .85).

#### Role-related and organizational factors

*Knowledge of breast and cervical cancer and screening* was measured using a 13-item scale developed by the NWP, with a score based on the percentage of correct answers (*α* = .62). *Self-efficacy* was measured using a 21-item scale validated among black LHAs [18] for measuring overall role self-efficacy and the sub-scales of growth self-efficacy, collective self-efficacy, and skills self-efficacy (response options, 1 (not at all confident) to 4 (very confident)) (*α* = .69–.85). *Leadership competence* was measured using the Sociopolitical Control Scale [70], with responses averaged to determine an overall score (*α* = .73). The 25-item Helper’s Perception Measure [59] measured mean perceptions of *role-related benefits and challenges* of being a LHA on a scale of 1 to 5 (1 = strongly disagree and 5 = strongly agree). *LHA role expectations (commitment and clarity)* were measured using nine items adapted for this study; participants indicated how much they agreed or disagreed with items using a 5-point scale (*α* = .86). *Job satisfaction as a LHA* was measured using a six-item adapted version of the validated Job Satisfaction Index [71] (*α* = .60). LHAs were asked if their current position with NWP was *paid* (yes; no). LHAs self-reported their *baseline activity level in NWP* as currently “active,” “inactive,” or “inactive but hope to become more active”. At the organizational level, we categorized LHAs as belonging to a site that was at an academic institution or had a *partnership or affiliation with an academic institution* (yes; no). Based on communication with the national and site leadership, this was defined as a NWP site that was housed within an academic institution or had a strong formal partnership with such an institution (e.g., schools of public health or medicine, academic hospitals, and cancer centers).

### Outcomes

Data on LHA-continued participation was collected about 18–24 months after the LHA baseline survey from project directors of sites where LHA participants were associated. Consistent with other studies [39, 41, 72], we examined levels of participation. We defined “activity level” based on whether LHAs had conducted two or more educational sessions over the past year (high/moderate activity) versus fewer than two sessions in the prior year (low/no activity). Two sessions were determined to be a meaningful level of participation based on the data distribution (the median number of programs per year was 2) and conversations with NWP project directors. We also examined LHA “retention” based on this same data; retention was defined as whether a trained volunteer LHA had conducted any sessions in the community in the previous year. Participants were categorized as “completely inactive” (0 sessions in the past year) or “retained” (at least one session in the past year).

### Statistical methods

Data were summarized using percentages, means, standard deviations, and ranges. To compare differences between LHAs who were retained (vs. completely inactive) and LHAs with high/moderate activity levels (vs. low/no activity levels), Fisher’s exact test or a chi-square test was used for categorical variables and analysis of variance (ANOVA) was used for continuous measures. To determine which variables remained independent predictors of activity level and retention status, multivariable logistic regression was used. All variables that were significant at the *p* ≤ 0.15 level in bivariate analysis were considered for inclusion in full multivariable logistic regression models. Models were then adjusted, using a stepwise selection approach, whereby only those variables that were significant at *p* < 0.05 were permitted to remain in the models; finally, reduced models are presented in Table 3. Results are organized according to the levels presented in our conceptual framework. We present models and tables in two separate ways (i.e., models that include and do not include baseline self-reported LHA activity) because of (1) concerns about the potential reliability of this self-report measure and (2) concerns about our ability to adequately explore other theoretically informed variables when baseline activity level is included in the model given the strength of this association and our sample size. Missing data was minimal. All analyses were performed using SAS 9.3 (SAS Institute, Cary, NC).

## Results

### LHA and position characteristics

Seventy-six (76) LHAs participated in this study; half of these women (50 %) (*n* = 38) were breast or cervical cancer survivors. LHAs were involved in the program for a mean of 65.8 months (approximately 5 1/2 years), ranging from 0 months (for newly trained LHAs) to 16 years. Ninety-two percent (92 %) of LHAs were in voluntary NWP positions (i.e., were reported not being paid a salary for being a LHA by NWP or the organization where NWP is based) and 51 % of the sample was not currently employed outside of the NWP (predominately due to retirement). Participating LHAs represented a diversity of geographic sites: 14 (18 %) were from Harlem, NY; 10 (13 %) were from Syracuse, NY; 17 (22 %) were from Little Rock, AR; 5 (7 %) were from Long Island, NY; 6 (8 %) were from Tampa, FL; 4 (5 %) were from Chicago, IL; 17 (22 %) were from Buffalo, NY; and 3 (4 %) were from Wichita, KS. Additional characteristics of LHAs, organized by activity level (high/moderate vs. low/no), are reported in Tables 1 and 2. Overall, 71 % of sites were affiliated with or had partnerships with academic institutions.

**Table 1:** Sociodemographic and health-related characteristics of lay health advisors (*n* = 76) by activity level

	All LHAs (*n* = 76)		Low/no activity (*n* = 28)		Highly/moderately active (*n* = 48)		p value
	*n* or mean (SD)	% or [range]	*n* or mean (SD)	% or [range]	*n* or mean (SD)	% or [range]	
Length of activity in program (months)	65.8 (53.0)	[0–192]	70.6 (55.5)	[3–192]	63.0 (51.9)	[0–180]	0.48
Level of activity at baseline							
Active	59	78	15	54	44	92	0.0005
Inactive	2	3	2	7	0	0	
Inactive, but hope to become more active	15	20	11	39	4	8	
Breast/cervical cancer survivorship							0.15
Cancer survivor	38	50	11	39	27	56	
No history of cancer	38	50	17	61	21	44	
Employed							0.15
Employed by the NWP	6	8	0	0	6	13	
Employed outside of the NWP	24	32	10	36	21	44	
Not employed/retired	29	51	18	64	21	44	
Position							0.08
Paid	6	8	0	0	6	13	
Voluntary	70	92	28	100	42	88	
Age	54.9 (13.5)	[21–78]	57.1 (12.9)	[22–78]	53.5 (13.9)	[21–76]	0.27
Education							0.31
≤Some college	30	39	13	46	17	35	
Associate’s or Bachelor’s degree	33	43	9	32	24	50	
Graduate or professional degree	13	17	6	21	7	15	
Income							0.45
<$10,000–$24,999	16	21	6	21	10	21	
$25,000–$49,999	22	29	7	25	15	31	
>$50,000	32	42	11	39	21	44	
Refused	6	8	4	14	2	4	
Marital status							0.45
Married	32	42	11	39	21	44	
Never married	23	30	10	36	13	27	
Separated/divorced/widowed	20	26	6	21	14	29	
Did not respond	1	1	1	4	0	0	
Have primary care provider	69	91	23	82	46	96	0.09
Primary insurance							0.45
Medicaid or Medicare	31	41	14	50	17	35	
Employer-provided insurance	33	43	10	36	23	48	
None/other	12	16	4	14	8	17	
Self-rated health							0.32
Excellent/very good	24	32	6	21	18	38	
Good	37	49	15	54	22	46	
Fair/poor	15	19	7	25	8	17	
Current smoker	6	8	3	11	3	6	0.66
Servings fruits and vegetables per day	3.1 (1.5)	[1–10]	2.8 (1.3)	[1–5]	3.2 (1.6)	[1–10]	0.29
Days per week of exercise	2.8 (2.2)	[0–7]	3.0 (2.6)	[0–7]	2.7 (2.0)	[0–7]	0.66

Program activity level was defined by the average number of educational sessions completed by LHAs per year. LHAs with low/no activity completed an average of less than two educational sessions per year and LHAs who were moderately/highly active completed two or more educational sessions per year.

**Table 2:** Individual, social, and role-related/organizational characteristics of lay health advisors (*n* = 76) by activity level

	All LHAs (*n* = 76)		Low/no activity (*n* = 28)		High/moderate activity (*n* = 48)		p value
Measure [possible range]	Mean score (SD)	Range	Mean score (SD)	Range	Mean score (SD)	Range	
Individual level factors							
Religiosity [9–36]	33.7 (3.2)	18–36	34.4 (2.3)	28–36	33.3 (3.6)	18–36	0.12
Racial pride [7–28]	24.2 (3.0)	10.0–28.0	24.1 (3.6)	10.0–28.0	24.2 (2.6)	18.0–28.0	0.92
Medical mistrust [1–5]	2.2 (0.8)	1.0–4.0	2.2 (0.9)	1.0–4.0	2.1 (0.8)	1.0–3.7	0.87
Discrimination in healthcare settings [0–3]	1.0 (1.2)	0–3	0.9 (1.3)	0–3	1.0 (1.2)	0–3	0.70
Basic psychological needs scale							
Autonomy [1–7]	6.1 (0.6)	4.0–7.0	6.0 (0.5)	4.6–6.9	6.1 (0.7)	4.0–7.0	0.64
Competence [1–7]	6.3 (0.8)	2.6–7.0	6.2 (1.0)	2.6–7.0	6.4 (0.7)	4.6–7.2	0.19
Relatedness [1–7]	6.4 (0.6)	4.4–7.0	6.3 (0.5)	5.1–7.0	6.2 (0.7)	4.4–7.0	0.82
Life engagement test [6–30]	28.8 (1.4)	24–30	28.8 (1.4)	24–30	28.9 (1.5)	24–30	0.76
Rosenberg self-esteem scale [6–24]	22.9 (1.5)	18–24	22.8 (1.5)	18–24	23.0 (1.5)	19–24	0.57
Social level factors							
Number of Social networks [0–12]	7.4 (1.7)	3–10	7.3 (1.6)	5–10	7.5 (1.7)	3–10	0.55
Total number of people in network [0—infinity]	50.5 (46.8)	13–272	44.0 (31.9)	14–146	54.2 (53.4)	13–272	0.37
Social provisions scale							
Overall [10–40]	37.5 (3.3)	26–40	37.5 (2.4)	32–40	37.5 (3.8)	26–40	0.99
Guidance [2–8]	7.7 (0.8)	4–8	7.7 (0.7)	5–8	7.7 (0.9)	4–8	1.00
Worth [2–8]	7.4 (0.9)	5–8	7.4 (0.8)	6–8	7.4 (1.0)	5–8	0.94
Integration [2–8]	7.4 (0.8)	5–8	7.2 (0.8)	5–8	7.5 (0.8)	5–8	0.23
Attachment [2–8]	7.5 (0.9)	4–8	7.6 (0.6)	6–8	7.4 (1.0)	4–8	0.46
Alliance [2–8]	7.6 (0.7)	5–8	7.7 (0.6)	6–8	7.6 (0.8)	5–8	0.71
Role-related and organizational factors							
Partnerships with other organizations							0.02
Academic	54	71	15	54	39	81	
Non-academic	22	29	13	46	9	19	
Breast cancer knowledge Mean score (% correct) [0–100]	84 (14)	46–100	79 (17)	46–100	88 (11)	54–100	0.008
Self-efficacy							
Overall [1–4]	3.7 (0.3)	2.8–4.0	3.6 (0.3)	2.8–4.0	3.8 (0.2)	3.1–4.0	0.04
Skills [1–4]	3.7 (0.3)	2.9–4.0	3.6 (0.3)	2.9–4.0	3.7 (0.3)	2.9–4.0	0.06
Growth [1–4]	3.7 (0.3)	2.9–4.0	3.6 (0.3)	2.8–4.0	3.8 (0.3)	2.9–4.0	0.06
Collective [1–4]	3.9 (0.3)	2.7–4.0	3.8 (0.4)	2.7–4.0	3.9 (0.3)	3.0–4.0	0.36
Leadership competence [1–5]	4.1 (0.5)	2.8–5.0	4.0 (0.5)	2.9–5.0	4.1 (0.5)	2.8–4.9	0.48
Helper’s perception							
Role benefits [1–5]	4.5 (0.4)	3.4–5.0	4.5 (0.4)	3.4–5	4.5 (0.4)	3.6–5.0	0.81
Role stressors [1–5]	1.6 (0.5)	1.0–3.0	1.7 (0.5)	1.0–2.6	1.6 (0.5)	1.0–3.0	0.55
Role expectations (clarity and commitment) [1–5]	4.6 (0.4)	3.2– 5.0	4.5 (0.5)	3.2–5.0	4.6 (0.4)	3.7–5.0	0.20
Job satisfaction index [1–5]	4.4 (0.5)	3.4–5.0	4.4 (0.5)	3.4–5.0	4.4 (0.5)	3.4–5.0	0.92

Program activity level was defined by the average number of educational sessions completed by LHAs per year. LHAs with low/no activity completed an average of less than two educational sessions per year and LHAs who were moderately/highly active completed two or more educational sessions per year

### Retention in the program and activity level

As reported by project directors, LHA retention in this sample was 68 %; 32 % (*n* = 24) of LHAs were completely inactive at follow-up (defined as conducting 0 sessions in the prior year). Of the 68 % who were still active at follow-up, 46 (88.5 %) reported being active at baseline, while the remaining 6 (11.5 %) reported that they were inactive but hoped to become more active. We found that 37 % (*n* = 28) of participants had low activity levels (defined as conducting fewer than two educational sessions in the past year) at follow-up. The number of sessions conducted per year by LHAs ranged from 0 to 32 (mean = 3.8; median = 2).

### Models predicting complete inactivity vs. retention in the program

Comparisons between LHAs who were completely inactive vs. retained (retention status at follow-up) showed possible relationships in bivariate models (at *p* ≤ 0.15) for variables at the individual and role-related/organizational levels. At the individual level, age (*p* = 0.06) and self-reported health (*p* = 0.12) were significant, with younger women and those reporting good to excellent health more likely to be retained. At the role-related and organizational level, a number of factors were significant: partnership with academic site (*p* = 0.002; academic sites had better retention); length in program (*p* = 0.13; less time in program for those who were retained, mean of 59.6 months vs. 79.4 months); breast cancer knowledge (*p* = 0.01; higher knowledge among retained); overall self-efficacy (*p* = 0.07) and growth self-efficacy (*p* = 0.08) (with higher self-efficacy for those retained); role clarity and commitment (*p* = 0.05) (with higher scores for those retained); and self-reported baseline LHA activity (*p* = 0.0005) (LHAs active at baseline were more likely to be retained).

#### Final models A1 and A2

Results from the final models (displayed in Table 3, models A1 and A2) showed that LHA retention was only significantly associated with variables from the role-related and organizational level. In model A1, LHAs based at *non-academic sites* had significantly decreased odds of being retained at follow-up than LHAs from academic sites (*p* = 0.003; odds ratio (OR), 0.16; confidence interval (CI), 0.05, 0.55). LHAs who had been in the *program longer* had decreased odds of being retained (*p* = 0.03; OR, 0.99; CI, 0.98, 0.99) than LHAs who were newer to the program. Finally, LHAs who reported greater role clarity and commitment had 5.7 times increased odds of being retained at follow-up than LHAs who had lower expectations or commitment (*p* = 0.01; OR, 5.73; CI, 1.43, 22.9). After adjustment for self-reported baseline LHA activity status (model A2), affiliation with a non-academic site remained significantly associated with decreased odds of LHA retention.

**Table 3:** Reduced multivariable models predicting lay health advisors’ retention in the program and high/moderate activity levels

Model A: model predicting retention^a^			
	Odds ratio	95 % confidence interval	p value
A1. Without baseline LHA activity			
Type of institution			
Academic	Reference	–	0.003
Non-academic	0.16	(0.05, 0.55)	
Length in program	0.99	(0.98, 0.99)	0.03
Role expectations (clarity and commitment)	5.73	(1.43, 22.9)	0.01
A2. Adjusting for baseline LHA activity			
Type of institution			
Academic	Reference	–	0.0005
Non-academic	0.022	(0.002, 0.19)	
Baseline activity level			
Active	62.7	(6.29, 579)	0.0003
Inactive	Reference	–	
Model B: model predicting high/moderate activity levels^b^			
	Odds ratio	95 % confidence interval	p value
B1. Without baseline LHA activity			
Type of Institution			
Academic	Reference	–	0.009
Non-academic	0.21	(0.06, 0.67)	
Self efficacy (overall)	12.7	(1.31, 123)	0.03
Religiosity	0.8	(0.64, 0.99)	0.05
B2. Adjusting for baseline LHA activity			
Type of Institution			
Academic	Reference	–	0.0002
Non-academic	0.05	(0.01, 0.24)	
Religiosity	0.70	(0.52, 0.95)	0.02
Baseline activity level			
Active	59.2	(9.56, 367)	<0.0001
Inactive	Reference	–	

^a^ Retention in program status was defined by whether an LHA conducted at least one educational sessions in the year. Retained LHAs conducted at least one educational session in the past year

^b^Program activity level was defined by the average number of educational sessions completed by LHAs in the year. LHAs considered to be moderately/highly active completed two or more educational sessions in the past year

### Models predicting high/moderate activity vs. low/no activity in the program

In bivariate models comparing LHAs who were highly/moderately active to those who were less/not at all active, possible differences (at *p* ≤ 0.15) were observed for variables under domains of individual level factors and role-related and organizational factors. Differences were observed for individual level factors: survivorship status (*p* = 0.15, active members more likely to be survivors), employment status (*p* = 0.15; active members more likely to be employed), having primary healthcare provider (*p* = 0.09, active members more likely to have PCP), and religiosity (*p* = 0.12, active members having lower scores). At the role-related and organizational level, the following were significant: partnership with academic site (*p* = 0.02, active members coming from academic sites), position payment (*p* = 0.08; active members more likely to be paid), breast cancer knowledge (*p* = 0.008; active members having higher scores), overall self-efficacy (*p* = 0.04), skill self-efficacy (*p* = 0.06), and growth self-efficacy (*p* = 0.06, active members having higher scores). Self-reported baseline activity status was also significantly associated with activity level, with LHAs active at baseline more likely to be active at follow-up (*p* = .0005).

#### Final models B1 and B2

In the final models for activity level (displayed in Table 3, models B1 and B2), LHA activity level was associated with variables at the individual and role-related/organizational levels. As seen in model B1, LHAs based at *non-academic sites* had decreased odds of being highly/moderately active compared to LHAs from academic sites (*p* = 0.009; OR, 0.21; CI, 0.06, 0.67), after controlling for self-efficacy and religiosity. In addition, higher overall *self-efficacy* scores were associated with increased odds of being highly/moderately active (*p* = 0.03; OR, 12.7; CI, 1.31, 123.00). Higher religiosity scores were associated with decreased odds of being highly/moderately active at follow-up (*p* = 0.05; OR, 0.80; CI, 0.64, 0.99). After adjustment for baseline activity status (model B2), affiliation with a non-academic site and religiosity remained associated with decreased odds of high/moderate activity level.

## Discussion

This study sought to address a notable gap in the literature by examining individual, social, and organizational/role-related factors that predict retention and activity level among a sample of 76 LHAs from eight urban and rural NWP sites. We found that 32 % of trained LHAs were completely inactive at follow-up, consistent with other studies that have been conducted in predominately low- and middle-income countries [39, 41, 48]. This study helps to quantify concerns about LHA retention and participation that have been reported elsewhere [35–38] and suggests that retention factors likely impact the sustainability of LHA programs. On the other hand, given that many of the LHAs were voluntary and not paid for participation in the program, the rates of retention and activity level are impressive and indicate strong commitment to the program among LHAs.

A notable finding from this study is that role-related and organizational factors were consistently associated with LHA retention and activity level. LHAs located at sites that were affiliated with or had strong partnerships with academic institutions were consistently more likely to be retained and to have high/moderate activity levels, even after adjustment for self-reported baseline LHA activity level. LHAs from non-academic sites had about an 80 % decrease in odds of being active, compared to those from academic sites (in models not adjusting for baseline LHA activity level). Though we are not aware of prior research that has empirically tested whether partnerships with academic institutions predict retention of LHAs, there is literature that suggests that such partnerships are integral to the sustainability of community-based programs, including LHA programs [34, 73, 74]. Based on this finding, we collected post hoc data from the project directors on differences between sites with and without academic partnerships that could contribute to these findings. Specifically, sites with academic partnerships were more likely to provide stipends to their LHAs, hold regular trainings, have a steering committee in place, and have physical space dedicated to the program (data not shown).

In addition, it is possible that sites with strong connections to academic institutions are more likely to have other sources of funding to support LHAs and LHA programs when there are interruptions in funding streams. This corresponds to findings from our earlier replication research [24] that demonstrated the crucial nature of having both “Administrative” and “Community Champions” for successful replication. The administrative champion would often be a key resource for securing support, grants and resources. It may be that the academic partners are more likely to have individuals serving as an administrative champion who are vested in shared outcomes with the LHAs, providing key organizational structure, resources and support to sustain such programs. Research is needed to understand this finding and to identify actionable processes in place at sites with academic partnerships to inform strategies that can be used to support and retain LHAs.

Other factors that impacted participation of LHAs in the program included length of time in NWP, suggesting that LHAs who have been in NWP longer may need extra support to prevent dropout or burn-out. Factors leading to and the impact of burn-out on LHAs and other volunteers are not well understood and warrant further research. Alternatively, as has been suggested in prior research in the context of Latina promotora programs [36, 37], LHA dropout may reflect the social mobility of LHAs who have gained new skills, competencies, and expertise and who are now are able to make advances in their careers. These explanations should be further explored in longitudinal research and qualitative or ethnographic research among LHAs. Additionally, in our research, LHAs who reported having clear role expectations were more than five times more likely to be retained (vs. completely inactive) at follow-up, suggesting that this is an important factor that impacts retention. This is consistent with other research that highlights the importance of role clarity, commitment, and expectations in influencing worker motivation and retention [39, 41, 49, 61]. This finding also suggests that clarifying role expectations when enrolling new LHAs is a critical part of the recruitment and training process that should be addressed on an ongoing basis.

In predicting activity level (high/moderate vs. low/no), we found that role self-efficacy was associated with greater activity level and that religiosity was associated with lower activity level in this sample. Consistent with prior research in global settings [46], this finding indicates that strategies to increase LHA self-efficacy through training and feedback may be important in increasing participation among LHAs. Furthermore, LHAs with high levels of religiosity may need additional support to be active in the program, potentially due to additional religious commitments affiliated with their faith and/or church duties or due to related volunteer responsibilities that may compete with their time. These factors should be further explored in larger studies, given that our observed association between self-efficacy and activity level had very wide confidence intervals, which, coupled with our sample size, suggests some instability in model estimates. Furthermore, our findings related to religiosity may be more specific to the NWP and other faith-based LHA programs, given that the program often includes faith-based elements and can be implemented in faith-based settings. Of note, role clarity and overall role self-efficacy were no longer associated with retention and activity level (respectively) after controlling for self-reported baseline LHA activity level. It is possible that self-efficacy is a marker of previous activity that in turn predicts subsequent activity, and this should be explored in future studies with larger sample sizes.

In contrast to the literature in global settings (e.g., [39, 41, 46, 48, 75]), we did not find that family approval/conflict, social prestige, and payment/economic incentives were critical factors impacting LHA participation. This suggests that it is important to consider differences in factors shaping LHA retention across different contexts. For example, economic and financial factors and family approval may be less important in the US context where LHAs may take on these roles in addition to other forms of paid employment and where women may have more economic and social independence from their families and partners [76]. These findings should also be interpreted in the context of our study, in which there was low variability in terms of payment because the overwhelming majority (92 %) of LHAs were unpaid volunteers. There is currently much debate about the payment of LHAs [77, 78] and the strengths and limitations of formalizing these roles. In addition, we had low variability on the items related to family conflict and social prestige (women reported low family conflict and high social prestige); these items were included as part of a larger scale that measured role challenges and benefits, respectively, and thus may contribute to differences across studies.

There are several limitations that should be recognized. While this is one of the largest studies to date of African American LHAs in the USA, it would be ideal to have a larger sample size for conducting quantitative analyses and multivariable models. As a result, our confidence intervals are wide for some analyses (e.g., self-efficacy), suggesting that the magnitude of the effect is difficult to determine given our sample size. Our findings are also primarily generalizable to LHA programs in community-based settings among racial/ethnic minority populations, including programs that involve cancer survivors as LHAs. While the characteristics of our sample are very similar to characteristics of other US-based community health workers (CHWs) [79], it is important to note distinctions between LHAs and CHWs. Despite similarities in characteristics and frequent overlap in qualifications and roles (e.g., provide culturally and linguistically appropriate health education, advocate for community and individual care), CHWs are typically paid and can have additional roles (e.g., proactively identifying and enrolling eligible individuals in health or social service programs, coordinating care for community residents) that may distinguish them from LHAs [80]. CHWs, but not LHAs, have been explicitly recognized under the Affordable Care Act as a part of the interdisciplinary training to support care through area health education centers and patient-centered medical homes [81, 82].

It is also possible that we underestimated LHA dropout, given that LHAs who participated in the study at follow-up may have already been more motivated and involved than those who did not participate. Ideally, we would have been able to conduct analyses based on scaling approaches (e.g., ordinal categories for activity level). Our sample size and the distribution of data prevented us from using this modeling approach; however, exploratory bivariate analyses using ordinal categories suggested that findings were consistent with those presented here. Unfortunately, we were not able to assess the performance or effectiveness of LHAs as part of our outcome, nor do we have information about the kind of payment that LHAs received (e.g., wage, stipend, etc.). In addition, due to limited sample size and the fact that there were eight sites, we did not have the statistical power to control for site in multivariable models. Since academic affiliation is significant and included in all models, this could control for some of the clustering of site. Finally, we recognize that sustainability and retention are likely affected by national, state, and local policies, including funding or financial resources of the sites, although these factors were beyond the scope of this study. Future research should examine broader policy factors that affect the sustainability of LHA programs.

Strengths of the study should be recognized. We conducted data collection from eight urban and rural sites in the USA and had a high response rate among LHAs. We conducted research among African American LHAs in the US context, a population and setting that has been highly underrepresented in this literature. Our results also contribute to limited research on the implementation and sustainability of evidence-based programs. Unlike many studies in this area, our study was longitudinal and the outcome was measured using an objective, non-self-reported measure (activity reported by project directors) with an excellent retention rate of participants. Finally, our conceptual framework was theory-based, comprehensive, and multi-level.

Very little research has tested strategies and interventions to promote involvement and activity levels among LHAs or the sustainability of LHA programs. The limited research that has been conducted suggests that a multi-level, localized, or context-specific approach may be important [38, 83]. Expanding incentive systems to include both financial and non-financial incentives may be important to motivate and retain LHAs [39, 41, 84, 85]. Stipends, gas cards, transportation, childcare, and meals have been suggested as strategies to increase participation and commitment among LHAs [35, 51]. Non-financial recognition (e.g., social and community recognition) may also be important in influencing participation [35–37, 39, 41, 60, 78], as are opportunities for career building and advancement (e.g., training, ongoing skill development) [5, 86].

We have additional recommendations, based on our findings. Clear communication about role expectations during recruitment and ongoing training to build and maintain self-efficacy may be important strategies to promote LHA retention, consistent with prior research [36, 37, 39, 41, 60, 86, 87]. Supportive feedback and supervision have been found to be important [5, 47, 60]. Forming partnerships and locating program champions (community members and professionals who take responsibility for identifying potential funding sources and network with other community partners and leaders to lobby for space, funding, and other resources) [36, 37] may also be critical for LHA program sustainability, as suggested by our research here and in our earlier work [24]. Building the capacity of *promotoras* and community participants to apply for funding [38] and building leadership capacity [88] are other strategies that have not been well-explored, but warrant further research. Given limited knowledge in this area, developing and testing evidence-based strategies and policies that promote sustainability of LHA programs should be a priority area for future research [38].

## Conclusions

This research highlights some of the key role-related and organizational factors that influence LHA program participation and retention, critical indicators of the sustainability of evidence-based LHA programs. This information can be used to inform implementation and sustainability of LHA programs for underserved populations in community settings and can help advance the development of conceptual frameworks related to overall sustainability of evidence-based programs. This study addresses an important gap, as there have been very few empirical studies that have provided insight into the sustainability of LHA programs in the US context. We suggest future directions for informing potential strategies to support LHAs (e.g., emphasis on role expectations and building self-efficacy at trainings) and for informing strategies to promote successful program implementation and sustainability (e.g., identifying program champions and developing partnerships with academic institutions). Given that LHA programs are increasingly being used globally and nationally to effectively improve health and address health disparities and that there are growing opportunities for the integration of LHAs in prevention and healthcare delivery, it is critical that more research focus on program implementation and sustainability to maximize their reach and impact.

## Abbreviations

AMEN: Annual Meeting for Education and Networking
CHW: community health worker
CI: confidence interval
LHA: lay health advisor
NWP: The National Witness Project
OR: odds ratio
